# HMGB3 promotes the malignant phenotypes and stemness of epithelial ovarian cancer through the MAPK/ERK signaling pathway

**DOI:** 10.1186/s12964-023-01172-7

**Published:** 2023-06-16

**Authors:** Hanlin Ma, Gonghua Qi, Fang Han, Panpan Gai, Jiali Peng, Beihua Kong

**Affiliations:** 1 Department of Obstetrics and Gynecology, Qilu Hospital of Shandong University, 107 Wenhua Xi Road, Jinan, 250012 China; 2 Gynecologic Oncology Key Laboratory of Shandong Province, Qilu Hospital of Shandong University, Jinan, 250012 China; 3 School of Medicine, Cheeloo College of Medicine, Shandong University, Jinan, 250012 China; 4 Department of Ophthalmology, Qilu Hospital of Shandong University, Jinan, 250012 China; 5 71217 of the Chinese People’s Liberation Army, Laiyang, 265200 China

**Keywords:** HMGB3, Ovarian cancer, Proliferation, Stemness, MAPK

## Abstract

**Background:**

Ovarian cancer, particularly epithelial ovarian cancer (EOC), is the leading cause of cancer-related mortality among women. Our previous study revealed that high HMGB3 levels are associated with poor prognosis and lymph node metastasis in patients with high-grade serous ovarian carcinoma; however, the role of HMGB3 in EOC proliferation and metastasis remains unknown.

**Methods:**

MTT, clonogenic, and EdU assays were used to assess cell proliferation. Transwell assays were performed to detect cell migration and invasion. Signaling pathways involved in HMGB3 function were identified by RNA sequencing (RNA-seq). MAPK/ERK signaling pathway protein levels were evaluated by western blot.

**Results:**

HMGB3 knockdown inhibited ovarian cancer cell proliferation and metastasis, whereas HMGB3 overexpression facilitated these processes. RNA-seq showed that HMGB3 participates in regulating stem cell pluripotency and the MAPK signaling pathway. We further proved that HMGB3 promotes ovarian cancer stemness, proliferation, and metastasis through activating the MAPK/ERK signaling pathway. In addition, we demonstrated that HMGB3 promotes tumor growth in a xenograft model via MAPK/ERK signaling.

**Conclusions:**

HMGB3 promotes ovarian cancer malignant phenotypes and stemness through the MAPK/ERK signaling pathway. Targeting HMGB3 is a promising strategy for ovarian cancer treatment that may improve the prognosis of women with this disease.

Video Abstract

**Supplementary Information:**

The online version contains supplementary material available at 10.1186/s12964-023-01172-7.

## Background

Ovarian cancer has been recognized as the fifth main cause of cancer deaths in the female population, especially among women over 40 years old [1, 2]. In 2022, approximately 57,090 new ovarian cancer cases and 24,494 ovarian cancer deaths were estimated to have occurred in China [3]. Epithelial ovarian cancer accounts for over 95% of ovarian malignant tumors, of which high-grade serous ovarian carcinoma (HGSOC) is the most common histologic subtype, accounting for approximately 80% of all ovarian cancer deaths [4]. More than 70% of patients with HGSOC are diagnosed at an advanced stage, due to ambiguous symptoms in the early disease stages [5]. Cytoreductive surgery combined with platinum-based doublet chemotherapy is the traditional treatment option for ovarian cancer [6]. Almost 70% of patients with advanced ovarian cancers are estimated to experience relapse and drug resistance after treatment [7]. Further, less than half of all patients bearing advanced-stage HGSOCs survive more than five years after diagnosis [8]. Despite the success of PARP inhibitors, there remains a lack of effective treatments for ovarian cancer. Therefore, there is a pressing need to clarify the molecular mechanisms underlying ovarian cancer progression and develop novel therapeutic targets.

High-mobility group box 3 (HMGB3; also known as HMG2A or HMG4) is a member of the high-mobility group protein family, which plays vital roles in DNA recombination, repair, and replication, and whose members act as cytokines to mediate responses to infection, injury, and inflammation [9]. HMGB3 was initially reported to be involved in regulating innate immune activity and differentiation of normal hematopoietic stem cell populations [10, 11]. HMGB3 is expressed at low levels in normal adult cells, but is often upregulated in tumor tissues, making it a promising target for therapeutic intervention [9]. Importantly, HMGB3 is an oncogene that promotes tumor occurrence, development, and chemotherapy resistance, through a variety of mechanisms, in breast cancer [12], colorectal cancer [13], thyroid cancer [14], neuroblastoma [15], nasopharyngeal carcinoma [16], and cervical cancer [17].

The mitogen-activated protein kinase (MAPK) signaling pathway is a major signal transduction pathway composed of cascades involving three kinases, namely MAPKKK, MAPKK, and MAPK [18]. The MAPK/ERK signaling pathway plays an important role in promoting cell survival and motility of multiple cancers, including ovarian cancer [19, 20]. HMGB3 is reported to promote drug resistance through regulating DNA damage response pathways in ovarian cancer [21, 22]. In our previous investigation, we found that HMGB3 is overexpressed in HGSOC tissues, and that high HMGB3 levels are associated with shorter overall survival and lymph node metastasis in patients with HGSOC [22], indicating that HMGB3 may have implications in ovarian cancer progression; however, the functions of HMGB3 in ovarian cancer proliferation and metastasis have not been effectively explored. It is also unclear whether MAPK/ERK signaling is involved in the functions regulated by HMGB3.

In the current study, we investigated the function of HMGB3 in ovarian cancer progression and further clarified the underlying mechanisms. We found that HMGB3 enhances ovarian cancer cell stemness, proliferation, and metastasis. Further, we clarified that the MAPK/ERK signaling pathway participates in HMGB3-mediated ovarian cancer malignant progression. These findings suggest that HMGB3 may be a promising target for development of therapeutic strategies against ovarian cancer.

## Methods

### Cell culture

SKOV3 cells were purchased from American Type Culture Collection (Manassas, VA, USA). A2780 cells were a kind gift from Jianjun Wei’s Laboratory. HEK293T cells were obtained from the Cell Bank of the Chinese Academy of Sciences (Shanghai, China). SKOV3, A2780, and HEK293T cells were cultured in McCoy’s 5 A medium, RPMI 1640 medium, and Dulbecco’s modified Eagle’s medium (DMEM), respectively, supplemented with 10% fetal bovine serum (FBS; Gibco, Grand Island, NY, USA) at 37 °C in 5% CO_2_. All cell lines were authenticated using short tandem repeat DNA profiling.

### Antibodies and reagents

Antibodies against HMGB3 (27465-1-AP), ETS-1 (12118-1-AP), MEK1/2 (11049-1-AP), CCND1 (60186-1-Ig), c-Myc (10828-1-AP), SOX2 (11064-1-AP), and ALDH1A1 (15910-1-AP) were purchased from Proteintech (Wuhan, China). Antibodies for p-MEK1/2 (9154), p-ERK1/2 (4370), and an Epithelial-Mesenchymal Transition Antibody Sampler Kit (9782) were obtained from Cell Signaling Technology (Danvers, MA, USA). Antibody against ERK1/2 (ab17942) was obtained from Abcam (Cambridge, UK). Antibody against β-actin (A5441) was purchased from Sigma-Aldrich (St. Louis, MO, USA). AZD6244 (S1008) and PD0325901 (S1036) were acquired from Selleck Chemicals (Houston, TX, USA).

### MTT assay

Ovarian cancer cell proliferation was assessed using the MTT assay. In brief, ovarian cancer cells were seeded into 96-well plates at a density of 1 × 10^3^ cells/per well and incubated for 1, 2, 3, 4, and 5 days. Cells were then stained with 20 µL of MTT reagent (5 mg/ml) for 2–4 h at 37 °C. Then, MTT reagent was discarded and 100 µL of DMSO was added to each well. Optical density values at 490 nm were measured using a microplate reader (Thermo Scientific, Waltham, MA, USA).

### Immunofluorescence staining

Cells (2 × 10^4^) were seeded into glass bottom culture dishes. After 24 h, cells were fixed with 4% paraformaldehyde for 15 min and blocked with normal goat serum for 30 min. Next, the cells were stained with anti-β-catenin (1:100) or anti-Vimentin (1:100) primary antibodies overnight, and then incubated with Alexa Fluor 488-conjugated goat anti-rabbit IgG secondary antibody (1:200) for 1 h. DAPI was used to stain nuclei. Images were acquired using a Sunny IRX-60 confocal microscope (Sunny Technology, Beijing, China).

### qRT–PCR

Total RNA samples were prepared using TRIzol reagent (15,596,018, Invitrogen). mRNA was subjected to reverse transcription with a PrimeScript RT Reagent Kit (RR037A, TaKaRa, Kyoto, Japan). Real-time PCR was performed using SYBR Premix Ex Taq (RR420A, TaKaRa) in an 7900HT Fast Real Time PCR System (Applied Biosystems, Waltham, MA, USA). The mRNA levels of specific genes were normalized to those of β-actin using the comparative Ct method (2^−ΔΔCt^). The primer sequences are provided in Additional file 1: Table S2.

### Western blot

Immunoblot analysis was performed using the following protocol, as described previously [23]. Cells were washed with PBS and lysed on ice in RIPA lysis buffer (Shanghai, China, P0013B, Beyotime) supplemented with 1 mM PMSF. Protein concentrations were determined using a BCA protein detection kit (P0012, Beyotime). Aliquots of whole cell lysates (30–50 µg per lane) were separated by SDS-PAGE and transferred onto PVDF membranes (Merck Millipore, Burlington, MA, USA). Then, the blots were sequentially incubated with 5% non-fat milk, primary antibodies (1:1000), and appropriate HRP-conjugated secondary antibodies (1:5000). Immunoreactive proteins were visualized using ECL reagent (ORT2655, PerkinElmer, Waltham, MA, USA). Images were acquired using a GE Amersham Imager 600 (GE, Chicago, IL, USA) and quantified with ImageJ 1.52a software.

### Colony formation assay

Cells (800–1000 per well) were seeded into 6-well plates and cultured for 1–2 weeks. Then, colonies were fixed with methanol and stained with crystal violet. The number of colonies (> 50 cells) was counted using ImageJ 1.52a software.

### Migration and invasion assay

Migration and invasion assays were performed in a 24-well transwell chambers system (353,097, BD Biosciences, Franklin Lakes, NJ, USA), with or without Matrigel (354,234, BD Biosciences), according to the manufacturer’s instructions. Briefly, 1–2 × 10^5^ cells were seeded in 200 µL medium without FBS into the upper chamber of transwell chambers, and 700 µL medium with 20% FBS was placed in the lower chambers. After culturing for an appropriate amount of time, invaded cells on the lower surface of membranes were fixed with methanol for 15 min, stained with crystal violet for 15 min, photographed at 200× magnification, and counted using ImageJ 1.52a.

### Plasmid construction and transfection

The pCMV-HMGB3 plasmid was generated by cloning the human *HMGB3* open reading frame into the pCMV vector (PS100069, OriGene, Rockville, MD, USA). HMGB3 shRNA-1 (TRCN0000018519) and shRNA-2 (TRCN0000018521) vectors were purchased from Sigma-Aldrich. ERK1/2 shRNA sequence is presented in Additional file 1: Table S1. All constructs were validated by DNA sequencing. For lentivirus packaging, psPAX2, pMD2.G, and target plasmid were co-transfected into HEK293T cells using Lipofectamine 2000 reagent (11668-019, Invitrogen). Supernatants containing virus particles were collected at 24 and 48 h after transfection. Collected virus supernatants were centrifuged at 3500 rpm for 10 min and filtered through a 0.45 μm filter. To obtain cells with HMGB3 stable overexpression or knockdown, cells were infected with lentivirus for 24 h and then selected for 1–2 weeks in a medium containing 2 µg/mL puromycin (P8833, Sigma-Aldrich).

### RNA sequencing

A2780 cells were transfected with HMGB3 siRNA or negative control siRNA for 48 h. HMGB3 siRNA sequence is shown in Additional file 1: Table S1. High-throughput mRNA-Seq was conducted and the resulting data were analyzed by Biomarker Technologies (Beijing, China).

### Sphere-formation

Adherent cells were washed with PBS, digested using trypsin, and maintained in serum-free DMEM/F12 medium supplemented with 1× B27, 20 ng/ml epidermal growth factor, and 20 ng/ml basic fibroblast growth factor. Then, 3000 cells were resuspended in 1:1 mixed Matrigel and DMEM/F12 medium. Cell suspensions were plated into ultra-low attachment 24-well culture plates, solidified at 37 °C for 15 min, and then 1 ml of DMEM/F12 medium added. Fresh medium was added every 3 days, and spontaneously generated spheroids were cultured for approximately 7 days.

### Cell cycle analysis

For cell cycle analysis, cells were washed with PBS, then fixed with cold 75% ethanol overnight at -20 °C. After rehydration with PBS, cells were stained with cell cycle detection buffer (G019-1-1, Jiancheng, Nanjing, China) for 30 min at 4 °C. A total of 1 × 10^4^ stained cells were acquired using a BD FACSCalibur flow cytometer instrument and analyzed with Modfit software.

### Animal experiments

Female BALB/c nude mice (4–6 weeks) were purchased from Gempharmatech Co., Ltd (Nanjing, China) and housed under specific pathogen-free conditions. Cells (5 × 10^6^) with HMGB3 knocked down or overexpressed were resuspended in PBS and then subcutaneously injected into the flanks of mice. Approximately two weeks post-injection, mice were euthanized and xenograft tumors removed for further analysis. Tumor volumes were calculated using the formula: volume = (length × width^2^)/2.

### EdU staining

Ovarian cancer cells (2000 cells/well) were seeded into 96-well plates and received the indicated treatments. Cells were stained with EdU reagent (10 µM) for 2 h at 37 °C. Then, cells were fixed using 4% paraformaldehyde for 15 min, followed by permeabilization with 0.5% Triton X-100 solution for 10 min. Next, the EdU signal was detected using a BeyoClick™ EdU-488 Kit (C0071S, Beyotime) with a fluorescence microscope. DAPI was used to stain nuclei.

### Statistical analysis

Experiments were repeated in triplicate and data are expressed as mean ± standard error of the mean (SEM). Student’s t-test or one-way ANOVA were used for statistical analysis with GraphPad Prism 9.0 (GraphPad Software, La Jolla, CA, USA). *P* < 0.05 was defined as statistically significant.

## Results

### HMGB3 facilitates ovarian cancer cell proliferation

**Fig. 1:**
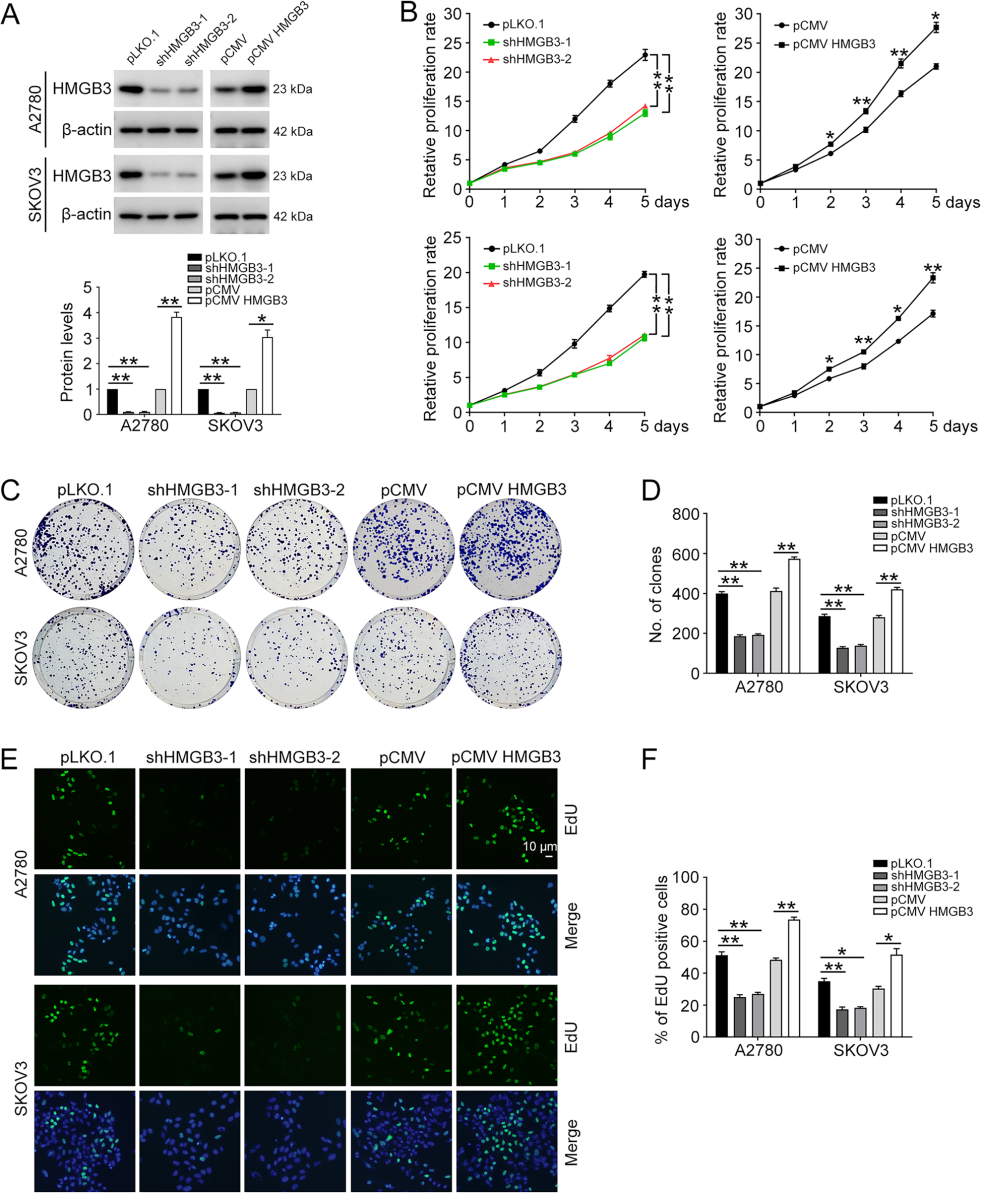
HMGB3 promotes ovarian cancer proliferation. **A** pLKO.1, HMGB3 shRNA-1 (shHMGB3-1), HMGB3 shRNA-2 (shHMGB3-2), pCMV, and pCMV HMGB3 plasmids were stably transfected into A2780 and SKOV3 cells. HMGB3 protein levels were determined by western blot. **B** Cells were seeded into 96-well plates and cultured for 1, 2, 3, 4, and 5 days, and an MTT assay was performed to assess cell viability. **C** A clonogenic assay was used to assess the colony formation efficiency of A2780 and SKOV3 cells with HMGB3 knocked down or overexpressed. **D** Quantification of the number of clones in **C**. **E** Proliferation of A2780 and SKOV3 cells with HMGB3 knocked down or overexpressed detected by EdU assay. Nuclei were stained using DAPI. Scale bar, 10 μm. **F** Quantification of the ratio of EdU positive cells in (**E**). Data are presented as the mean ± SEM, *p < 0.05, **p < 0.01, n = 3

Our previous report demonstrated that HMGB3 is highly expressed in patients with HGSOC and that high HMGB3 expression is positively correlated with shorter overall survival [22]; however, the function of HMGB3 in ovarian cancer proliferation remains unclear. To clarify the role of HMGB3 in ovarian cancer cell proliferation, A2780 and SKOV3 cells with HMGB3 stably overexpressed or knocked down were constructed using lentiviral vectors. The knockdown and overexpression efficiency of HMGB3 were verified using western blot analysis (Fig. 1A). MTT assay showed that HMGB3 knockdown inhibits ovarian cancer cell proliferation, while HMGB3 overexpression significantly promoted the proliferation of these cells (Fig. 1B). Consistently, the results of a clonogenic assay also demonstrated that HMGB3 increases the colony formation ability of ovarian cancer cells (Fig. 1C and D). Additionally, the EdU assay showed that HMGB3 overexpression clearly enhances the A2780 and SKOV3 cell proliferation, whereas HMGB3 knockdown slows cell proliferation (Fig. 1E and F). Moreover, flow cytometry analysis showed that HMGB3 knockdown induces G2/M phase arrest compared with the control group (Additional file 1: Fig. S1). Thus, our data suggest that HMGB3 promotes ovarian cancer cell proliferation.

### HMGB3 promotes ovarian cancer cell mobility

**Fig. 2:**
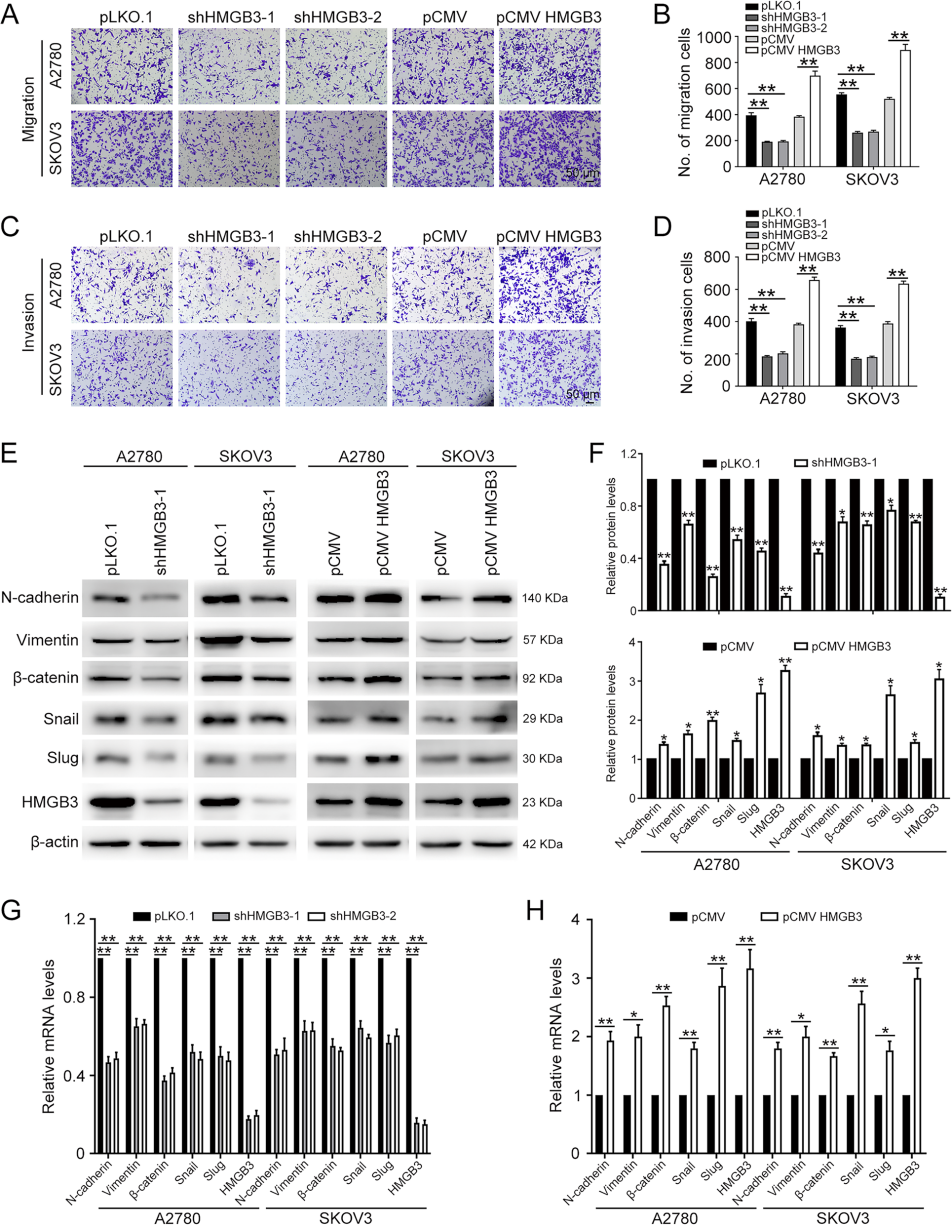
HMGB3 promotes ovarian cancer cell migration and invasion. **A** The effects of HMGB3 knockdown or overexpression on A2780 and SKOV3 cell migration determined by transwell assay. **B**) uantification of numbers of migration cells in (**A**). **C** The effects of HMGB3 knockdown or overexpression on invasion of A2780 and SKOV3 cells determined by transwell assay. Scale bar, 50 μm. **D** Quantification of numbers of invasion cells in (**C**). **E** Epithelial-mesenchymal transition -related markers in A2780 and SKOV3 cells with HMGB3 knockdown or overexpression detected by western blot. **F** Quantification of the protein levels in (**E**). qRT-PCR detection of epithelial-mesenchymal transition-related markers in A2780 and SKOV3 cells with HMGB3 knocked down (**G**) or overexpressed (**H**). Data are presented as the mean ± SEM, *p < 0.05, **p < 0.01, n = 3

As high HMGB3 expression is associated with lymph node metastasis in patients with HGSOC [22], we hypothesized that HMGB3 plays a role in ovarian cancer cell migration and invasion. Transwell assays were conducted to investigate metastasis of ovarian cancer cells with HMGB3 overexpressed or knocked down. The results demonstrated that HMGB3 inhibition impairs A2780 and SKOV3 cell migration and invasion, while HMGB3 overexpression have the opposite effects (Fig. 2A–D). Western blot, immunofluorescence analysis, and qRT-PCR were performed to examine the expression of epithelial-mesenchymal transition markers and demonstrated that HMGB3 knockdown lead to reduced expression of mesenchymal markers, including N-cadherin, vimentin, β-catenin, snail, and slug. Correspondingly, the expression of these mesenchymal markers was upregulated in cells with HMGB3 overexpression (Fig. 2E–H, Additional file 1: Fig. S2). These results suggest that HMGB3 enhances ovarian cancer cell migration and invasion.

### HMGB3 regulates stem cell pluripotency and the MAPK signaling pathway

**Fig. 3:**
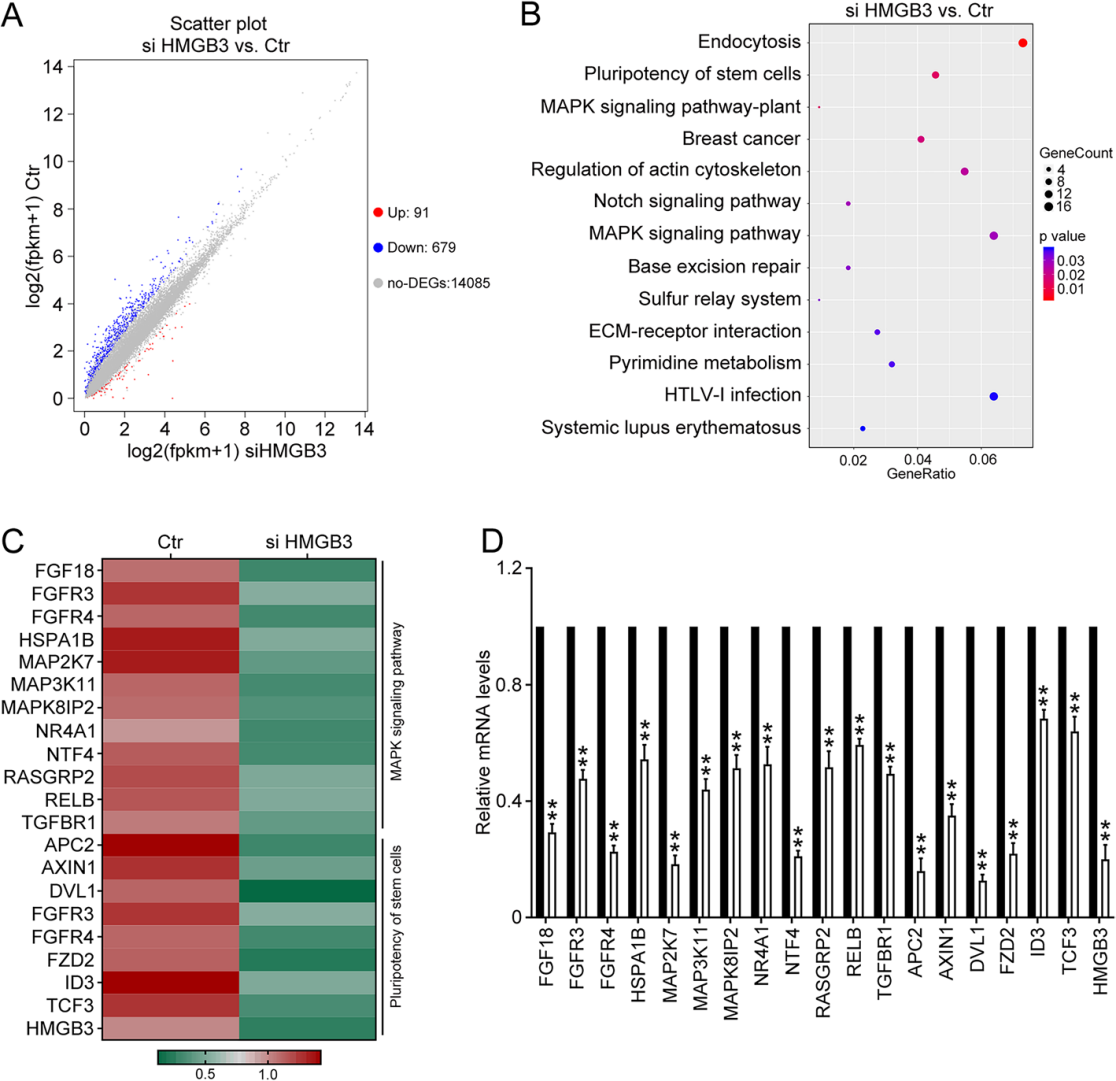
RNA sequencing analysis of signaling pathways involved in HMGB3 function. A2780 cells were transfected with HMGB3 siRNA (siHMGB3) or negative control siRNA (Ctr) for 48 h. High-throughput RNA sequencing analysis was used to compare mRNA expression profiles of the siHMGB3 and Ctr groups. **A** Volcano plot showing differentially expressed genes (DEGs) between the siHMGB3 and Ctr groups. In total, 91 genes were up-regulated (Up) and 679 genes were down-regulated (Down); other genes expression levels were not significantly altered (no-DEGs) (**B**) Kyoto Encyclopedia of Genes and Genomes enrichment analysis of genes down-regulated in the siHMGB3 group relative to the Ctr group. **C** Heatmap showing genes involved in stem cell pluripotency and the MAPK signaling pathway down-regulated in the siHMGB3 group relative to the Ctr group. **D** A2780 cells were transfected with HMGB3 siRNA (siHMGB3) or negative control siRNA (Ctr) for 48 h, and qRT-PCR used to verify down-regulation of representative genes in the siHMGB3 group relative to the Ctr group. Data are presented as the mean ± SEM, *p < 0.05, **p < 0.01, n = 3

To clarify the mechanism by which HMGB3 promotes the malignant progression of ovarian cancer, RNA-seq was applied to investigate the signaling pathways influenced by HMGB3 knockdown. Following transient transfection of HMGB3 siRNAs into A780 cells for 48 h, high-throughput sequencing identified 91 upregulated and 679 downregulated genes between the HMGB3 knockdown (siHMGB3) and control (Ctr) groups (Fig. 3A). Kyoto Encyclopedia of Genes and Genomes pathway analysis of down-regulated genes showed that stem cell pluripotency and the MAPK signaling pathway, which play important roles in tumor progression, are strongly enriched in the HMGB3 knockdown group (Fig. 3B and C). Then, we conducted qPCR to verify the expression of key genes involved in stem cell pluripotency and the MAPK signaling pathway identified by RNA-seq (Fig. 3D). These data indicate that HMGB3 may regulate the malignant phenotypes of ovarian cancer by influencing stem cell pluripotency and MAPK signaling.

### HMGB3 activates the MAPK/ERK signaling pathway in ovarian cancer

**Fig. 4:**
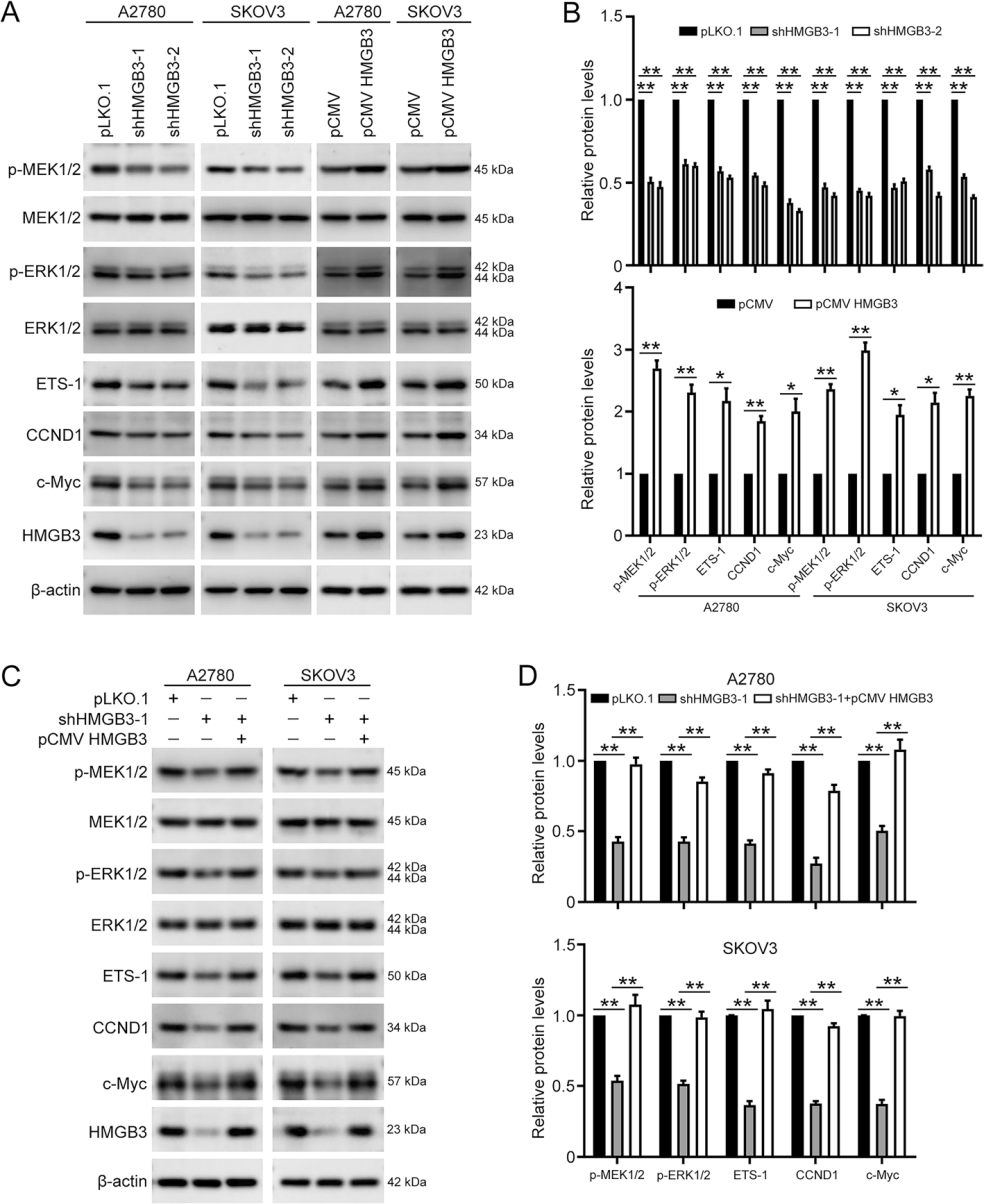
HMGB3 activates the MAPK/ERK signaling pathway in ovarian cancer cells. **A** p-MEK1/2, MEK1/2, p-ERK1/2, ERK1/2, ETS-1, CCND1, c-Myc, HMGB3, and β-actin protein levels of in A2780 and SKOV3 cells with HMGB3 knocked down or overexpressed detected by western blot. **B** Quantification of the protein levels in (**A**). **C** Protein levels in ovarian cancer cells transfected with pLKO.1, HMGB3 shRNA-1 (shHMGB3-1) and/or pCMV HMGB3 detected by western blot. (D) Quantification of the protein levels in (**C**). Data are presented as the mean ± SEM, *p < 0.05, **p < 0.01, n = 3

Next, to confirm whether HMGB3 activates the MAPK signaling pathway in ovarian cancer, western blot was used to examine the phosphorylation levels of p-MEK1/2 and p-ERK1/2, which are key factors in the MAPK/ERK pathway. Our results demonstrated that HMGB3 overexpression increases p-MEK1/2 and p-ERK1/2 levels, which were inhibited by HMGB3 knockdown (Fig. 4A and B). Our data also showed that HMGB3 suppression reduces the expression of major downstream target genes of the MAPK/ERK signaling pathway, including ETS-1, CCND1, and c-Myc, which have key roles in ovarian cancer occurrence and development. Consistently, HMGB3 overexpression promoted the expression of these three factors (Fig. 4A and B). Moreover, the decreased MAPK/ERK activity caused by HMGB3 knockdown was ameliorated by restoring HMGB3 expression (Fig. 4C and D). Thus, our data prove that HMGB3 activates the MAPK/ERK signaling pathway in ovarian cancer cells. Hence, MAPK/ERK signaling may contribute to HMGB3-induced malignant progression of ovarian cancer.

### HMGB3 promotes ovarian cancer stemness through MAPK/ERK signaling

**Fig. 5:**
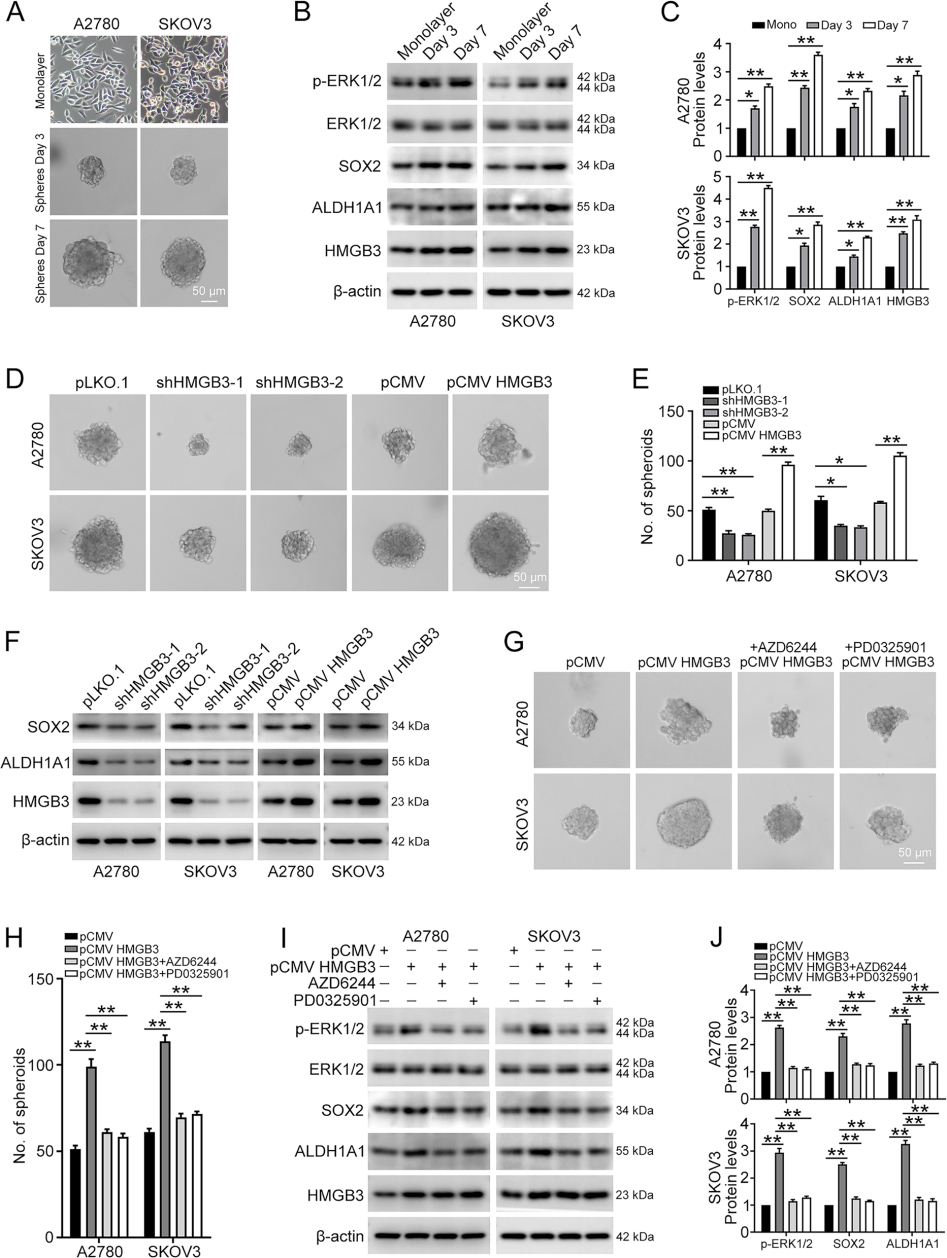
HMGB3 enhances ovarian cancer stemness through the MAPK/ERK signaling pathway. A2780 and SKOV3 cells were cultured in semi-solid serum-free medium for 0, 3, and 7 days. **A** Representative morphology of spheroids derived from A2780 and SKOV3 cells. Parental cells (monolayer); day 3 spheroid cells derived from parental cells (day 3); day 7 spheroid cells derived from parental cells (day 7). Scale bar, 50 μm. **B** p-ERK1/2, ERK1/2, SOX2, ALDH1A1, HMGB3, and β-actin protein levels in monolayer (Mono) and spheroid cells detected by western blot. **C** Quantification of the protein levels in (**B**). A2780 and SKOV3 cells with HMGB3 knocked down or overexpressed were cultured in semi-solid serum-free medium for 7 days. **D** The number and volume of spheroids formed were determined via microscopy; representative pictures are shown. Scale bar, 50 μm. (E) Quantification of the number of spheroids in (**D**). **F** SOX2, ALDH1A1, HMGB3, and β-actin protein levels in spheroids detected by western blot. Quantification of the protein levels is shown in Additional file 1: Fig. S3. **G** A2780 and SKOV3 cells with HMGB3 overexpression were cultured in semi-solid serum-free medium for 7 days with or without MAPK signaling pathway inhibitor (AZD6244, 1 µM; PD0325901, 2 µM) treatment. The number and volume of spheroids formed were determined via microscopy, and representative pictures are shown. **H** Quantification of the number of spheroids in (**G**). **I** p-ERK1/2, ERK1/2, SOX2, ALDH1A1, HMGB3, and β-actin protein levels in spheroids detected by western blot. (J) Quantification of the protein levels in (**I**). Data are presented as the mean ± SEM, *p < 0.05, **p < 0.01, n = 3

Since RNA-seq indicated that HMGB3 mediates stem cell pluripotency and MAPK signaling, we investigated whether HMGB3 plays a role in maintaining ovarian cancer cell stemness. A2780 and SKOV3 cells were cultured in a semi-solid serum-free medium to form spheroid clusters (Fig. 5A). The protein levels of SOX2 and ALDH1A1, two stem-related markers, were upregulated during the process of spheroid formation. We observed that HMGB3 protein expression gradually increased when adherent cells became suspensive, denser, and multi-cellular spheroids. Further, MAPK/ERK signaling was also activated during this progress, implying that HMGB3 and MAPK/ERK have important roles in modulating stem-like properties of ovarian cancer cells (Fig. 5B and C). Next, cells with HMGB3 knocked down or overexpressed were maintained in serum-free medium for 7 days to form spheroids. The results showed that HMGB3 knockdown significantly impairs the spheroid forming ability of ovarian cancer cells and inhibits SOX2 and ALDH1A1 expression. In contrast, HMGB3 overexpression enhanced the spheroid forming ability of cells and increased SOX2 and ALDH1A1 expression levels (Fig. 5D–F). The MAPK/ERK signaling pathway is reported to positively regulate cancer stemness [24, 25]; therefore, we explored whether MAPK/ERK is involved in HMGB3-induced increased ovarian cancer cell stemness. The results showed that HMGB3 overexpression fails to strengthen the spheroid forming ability of cells and increase stem-related marker levels in the presence of MAPK/ERK inhibitors (Fig. 5G–J). Moreover, the effects of HMGB3 overexpression were greatly reduced in cells with ERK1/2 knocked down (Additional file 1: Fig. S4). Therefore, our results suggest that HMGB3 facilitates stem-like characteristics in ovarian cancer cells through activating MAPK/ERK signaling.

### HMGB3 enhances ovarian cancer proliferation and mobility via the MAPK/ERK signaling pathway

**Fig. 6:**
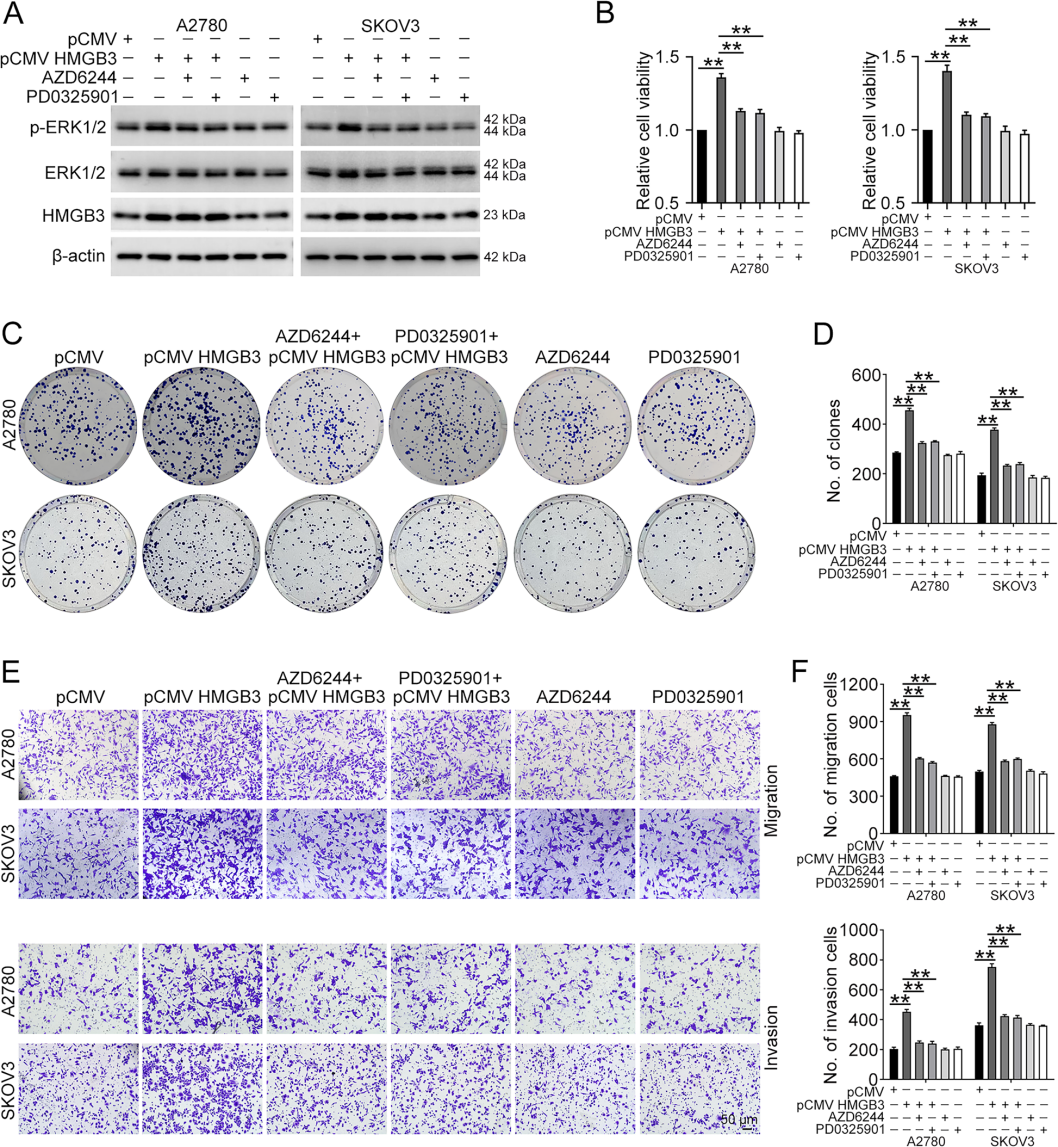
HMGB3 promotes the malignant phenotypes of ovarian cancer via the MAPK/ERK signaling pathway. **A** A2780 and SKOV3 cells with HMGB3 overexpression were seeded into 6 cm dishes and then treated with or without AZD6244 (5 µM)/PD0325901 (10 µM) for 24 h. p-ERK1/2, ERK1/2, HMGB3, and β-actin protein levels detected by western blot. **B** A2780 and SKOV3 cells with HMGB3 overexpression were seeded into 96-well plates and then treated with or without AZD6244 (5 µM)/PD0325901 (10 µM) for 72 h. Cell viability was detected by MTT assay. **C** A2780 and SKOV3 cells with HMGB3 overexpression were treated with or without AZD6244 (1 µM)/PD0325901 (2 µM) for 1–2 weeks. Colony formation efficiency was assessed by clonogenic assay. **D** Quantification of the number of clones in (**C**). **E** A2780 and SKOV3 cells with HMGB3 overexpression were seeded into transwell plates and then treated with or without AZD6244 (5 µM)/PD0325901 (10 µM) for 12–24 h. Cell migration and invasion were evaluated by transwell assay. Scale bar, 50 μm. **F** Quantification of the number of cells in (**E**). Data are presented as the mean ± SEM, **p < 0.01, n = 3

To investigate whether the MAPK/ERK signaling pathway is required for HMGB3-mediated proliferation and mobility of ovarian cancer, we used two specific MAPK signaling pathway inhibitors, AZD6244 and PD0325901, to suppress MAPK/ERK activity. The results of western blot confirmed that MAPK/ERK signaling is inhibited in the presence of AZD6244 or PD0325901 (Fig. 6A). As shown in Fig. 6B, MTT assays demonstrated that HMGB3 overexpression promotes ovarian cancer cell proliferation, while AZD6244 or PD0325901 administration abrogates this effect. Clonogenic assays also showed that MAPK/ERK inhibitors blocks the enhanced proliferation evoked by HMGB3 overexpression (Fig. 6C and D). Blocking MAPK/ERK signaling using AZD6244 or PD0325901 also reversed the increased migration and invasion abilities of ovarian cancer cells induced by HMGB3 overexpression (Fig. 6E and F). Moreover, HMGB3 overexpression failed to enhance the proliferation and metastasis abilities of ovarian cancer cells with ERK1/2 knocked down (Additional file 1: Fig. S5). Hence, HMGB3 promotes ovarian cancer malignant phenotypes through activating the MAPK/ERK signaling pathway.

### HMGB3 promotes ovarian cancer proliferation in vivo

**Fig. 7:**
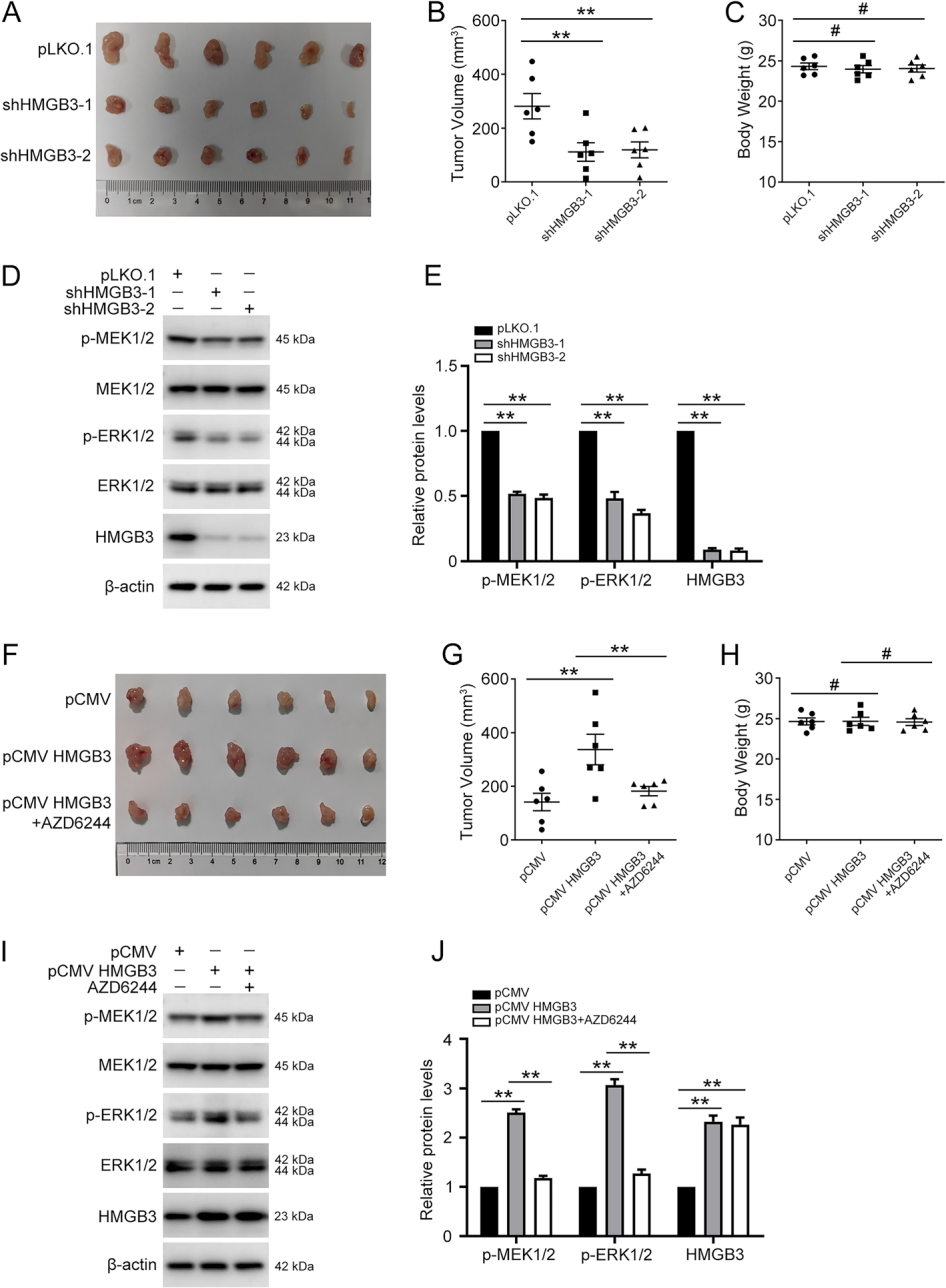
HMGB3 promotes ovarian cancer cell proliferation in vivo through the MAPK/ERK signaling pathway. A2780 cells (5 × 10^6^) transfected with pLKO.1, HMGB3 shRNA-1 (shHMGB3-1), or HMGB3 shRNA-2 (shHMGB3-2) were subcutaneously injected into nude mice. Mice were divided into three groups: pLKO.1 (Ctr), shHMGB3-1, and shHMGB3-2 (n = 6 per group). Two weeks post-injection, mice were euthanized and the xenograft tumors were removed. **A** Tumors from each group are shown. **B** Tumor volumes of each group. **C** Body weight of each group. **D** p-MEK1/2, MEK1/2, p-ERK1/2, ERK1/2, HMGB3, and β-actin protein levels in tumor tissues detected by western blot. **E** Quantification of the protein levels in (**D**). A2780 cells (5 × 10^6^) transfected with pCMV or pCMV HMGB3 were subcutaneously injected into nude mice. Mice were divided into three groups: pCMV (Ctr), pCMV HMGB3, and pCMV HMGB3 + AZD6244 (n = 6 per group). One group of mice received an intraperitoneal injection of AZD6244 (25 mg/kg) once a day. Two weeks post-injection, mice were euthanized and xenograft tumors were removed. **F** Tumors from each group are shown. **G** Tumor volumes of each group. **H** Body weight of each group. **I** p-MEK1/2, MEK1/2, p-ERK1/2, ERK1/2, HMGB3, and β-actin protein levels in tumor tissues detected by western blot. **J** Quantification of the protein levels in (**I**). Data are presented as mean ± SEM, ^#^*p* > 0.05, **p < 0.01, n = 6

Next, we investigated the effects of HMGB3 and MAPK/ERK signaling on ovarian cancer proliferation in a xenograft model. A2780 cells transfected with pLKO.1 (Ctr), HMGB3 shRNA-1 (shHMGB3-1), or HMGB3 shRNA-2 (shHMGB3-2) were intraperitoneally injected into BALB/c nude mice to generate xenograft models. As shown in Fig. 7A–C, tumor volumes were significantly smaller in mice injected with HMGB3 shRNA cells than in those injected with control pLKO.1 cells. Consistent with the results of in vitro experiments, HMGB3 knockdown inhibited MAPK/ERK signaling pathway activation in vivo (Fig. 7D and E). In contrast, HMGB3 overexpression dramatically increased tumor volumes (Fig. 7F–H; Additional file 1: Fig. S6) and activated MAPK/ERK signaling (Fig. 7I and J) relative to the control group, whereas administration of the MAPK/ERK signaling pathway inhibitor, AZD6244, or ERK1/2 knockdown, reversed the effect of HMGB3 overexpression. Thus, our findings indicate that HMGB3 effectively promotes ovarian cancer proliferation in vivo.

## Discussion

The high mortality associated with ovarian cancer is primarily attributable to asymptomatic tumor growth, which results in most patients being diagnosed at advanced stages of the disease [26]. Further, most patients will relapse after surgery and standard platinum/taxane-based chemotherapy [27]. Therefore, there is an urgent need to develop new therapeutic strategies. In the current study, our results demonstrated that HMGB3, a well-known oncogene, promotes the malignant progression of ovarian cancer through activating the MAPK signaling pathway in vitro and in vivo, which may inform the development of new targeted therapy strategies for ovarian cancer.

HMGB3 is aberrantly expressed in a number of malignancies, contributing to tumor cell progression and predicting poor outcomes [9]. In our previous study, we found that high HMGB3 expression predicted poor prognosis and lymph node metastasis in patients bearing HGSOC. Further, HMGB3 promotes PARP inhibitor resistance of ovarian cancer through directly interacting with PARP1 [22]. In this study, we further investigated the function of HMGB3 in ovarian cancer. Our data demonstrated that HMGB3 overexpression promotes ovarian cancer proliferation, migration, invasion, and stemness, which could be inhibited by HMGB3 knockdown. Our findings add to understanding of ovarian cancer pathogenesis and provide new therapeutic targets for this disease.

HMGB3 has been demonstrated to regulate several carcinogenic signaling pathways in multiple types of cancer [9]. HMGB3, regulated by miR-216a, promotes esophageal cancer cell growth through enhancing Wnt/β-catenin pathway activity [28]. Moreover, HMGB3 promotes tumor development by regulating Wnt/β-catenin signaling in cervical cancer [17], non-small cell lung cancer [29], and colorectal cancer [30]. HMGB3 inhibition inhibits colony formation and induces apoptosis by increasing reactive oxygen species accumulation and decreasing MMP, p-mTOR, and STAT3 levels in human breast cancer cells [12]. In this study, RNA-Seq revealed that HMGB3 may be involved in MAPK signaling regulation. The MAPK pathway influences almost all aspects of life and has numerous substrates involved in execution of specific cell fate decisions in response to extracellular signals [31]. The MAPK signaling cascade is invariably altered in malignant cancers and has implications in tumor occurrence, development, and drug resistance [32]. Our data proved that HMGB3 activates MAPK/ERK signaling in ovarian cancer, as indicated by increased MEK1/2 and ERK1/2 phosphorylation, and the upregulation of its downstream molecules, including ETS-1, CCND1, and c-Myc. Inhibition of MAPK/ERK signaling pathway using specific inhibitors reversed the enhanced proliferation, metastasis, and stemness caused by HMGB3 overexpression. Thus, MAPK signaling inhibition by targeting HMGB3 may serve as a novel strategy for ovarian cancer therapy.

Cancer stem cells (CSCs) are a small proportion of cells within tumors with distinct phenotypes and a high tumorigenic potential [33]. CSCs express high levels of stemness-associated factors and are considered as the origin of tumor recurrence, drug resistance, and relapse [34]. Thus, CSCs represent an optimal therapeutic target to tackle ovarian cancer. HMGB3 is proven to mediate cancer cell stemness in many malignancies. LINC00319 stimulates CD133^+^CD144^+^ TU177 cell self-renewal ability and tumorigenicity by upregulating HMGB3 via recruitment of E2F1 in laryngeal squamous cell carcinoma [35]. HMGB3 cooperates with SOX9 to induce NANOG transactivation, promote the expression of oncogenic genes downstream of NANOG, and further enhance prostate adenocarcinoma cell survival and migration [36]. In the current study, we demonstrated that HMGB3 also positively regulates ovarian cancer stemness. HMGB3 overexpression led to elevated expression of stemness-associated markers and strengthened ovarian cancer spheroid formation ability, while HMGB3 knockdown had the opposite effect. Moreover, our findings show that MAPK signaling is involved in HMGB3-mediated stemness regulation.

## Conclusions

**Fig. 8:**
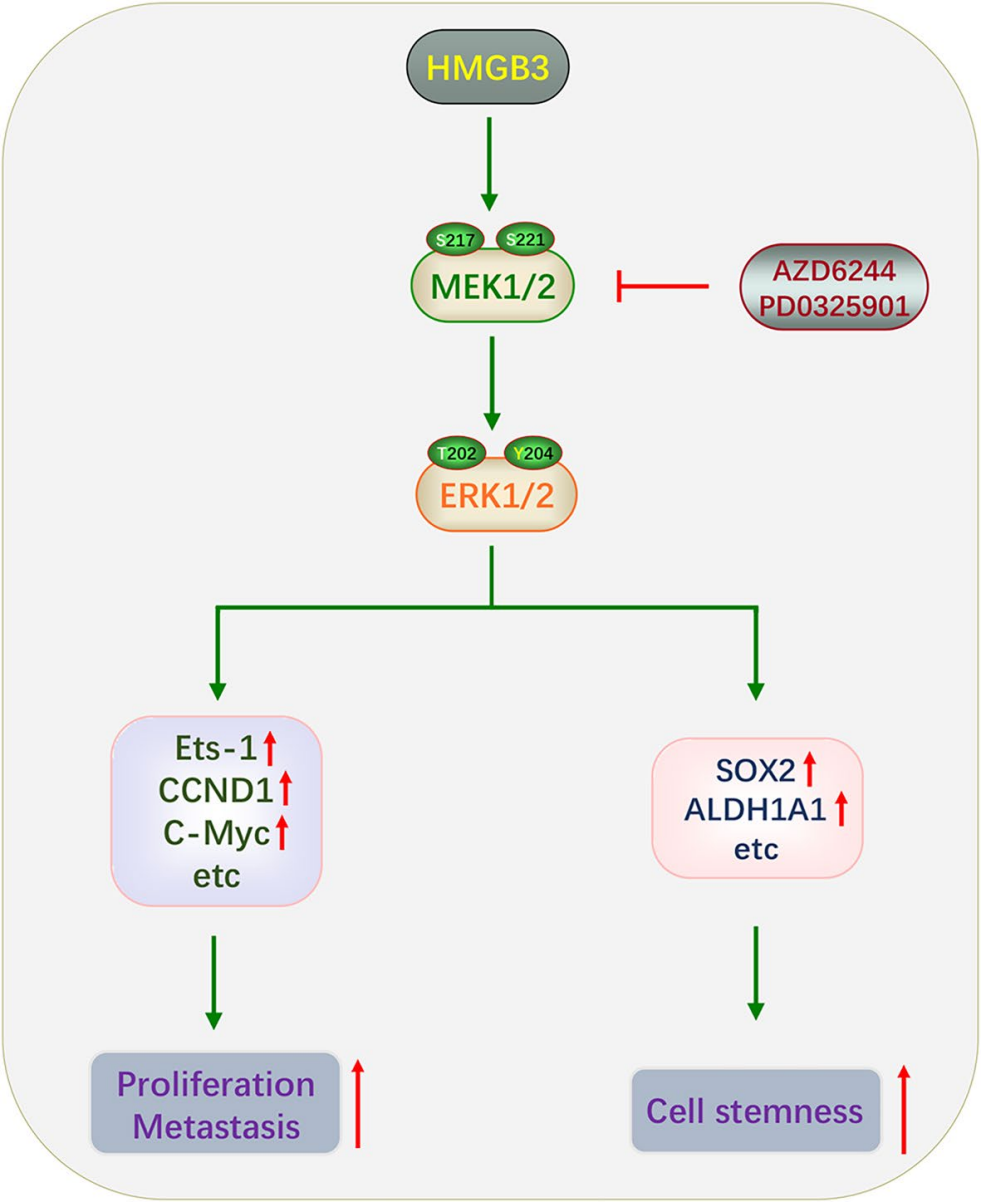
Schematic summary of the study findings. HMGB3 overexpression activates the MAPK/ERK signaling pathway, thereby promoting ovarian cancer proliferation, metastasis, and stemness. Inhibition of the MAPK/ERK signaling pathway using specific inhibitors counteracts the effects of HMGB3

In conclusion, our findings show that HMGB3 promotes ovarian cancer proliferation, mobility, and stemness. Mechanistically, HMGB3 facilitated the activation of MAPK/ERK signaling in ovarian cancer. We further demonstrated that MAPK/ERK signaling inhibition counteracts the effect of HMGB3 overexpression (Fig. 8). These findings indicate that HMGB3 is a promising target to develop therapeutic strategies against ovarian cancer.

## Supplementary Information

**Additional file 1.**

## Data Availability

All data generated or analyzed during this study are included in this published article [and its supplementary information files].

## Abbreviations

CSCs: Cancer stem cells
DMEM: Dulbecco’s modified Eagle’s medium
FBS: Fetal bovine serum
HGSOC: High-grade serous ovarian carcinoma
HMGB3: High mobility group box 3
MAPK: Mitogen-activated protein kinase
RNA-seq: RNA sequencing
