# Supplementary material for: HMGB3 promotes the malignant phenotypes and stemness of epithelial ovarian cancer through the MAPK/ERK signaling pathway
Source: Cell Commun Signal. 2023 Jun 16;21:144. doi: 10.1186/s12964-023-01172-7 (PMC10273509; doi:10.1186/s12964-023-01172-7)
Supplement: Supplementary file 2 — Additional file 1. [file 12964_2023_1172_MOESM1_ESM.docx]

**Supplementary Figures**


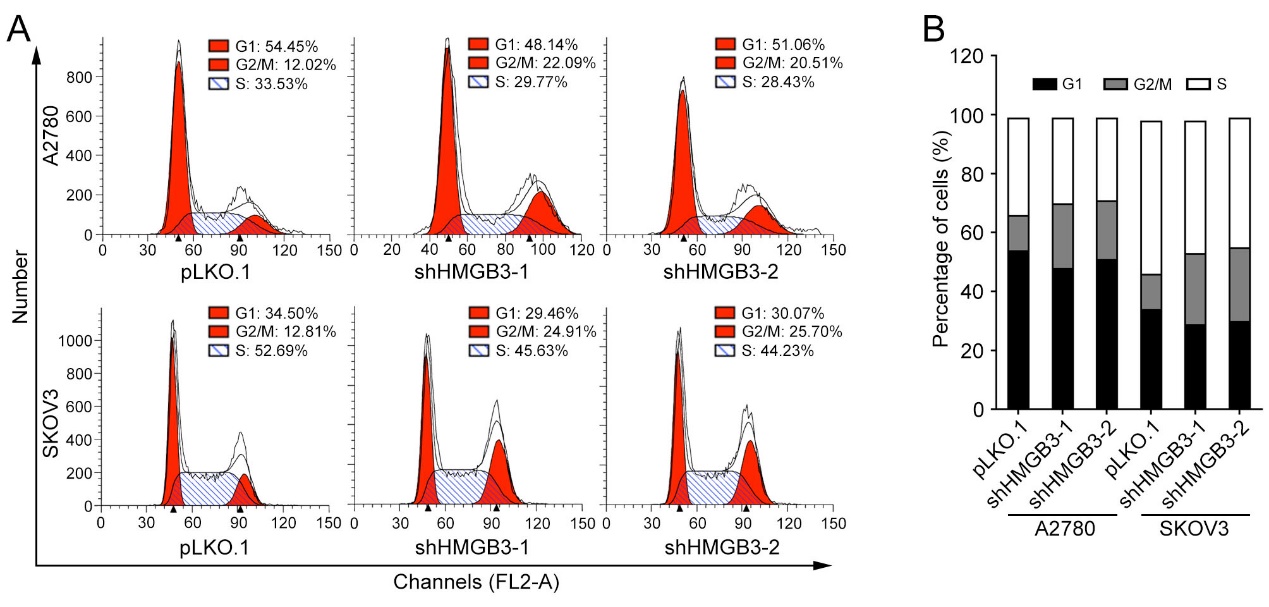


**Figure S1.** Cell cycle distribution was analyzed by flow cytometry in ovarian cancer cells with HMGB3 knockdown.


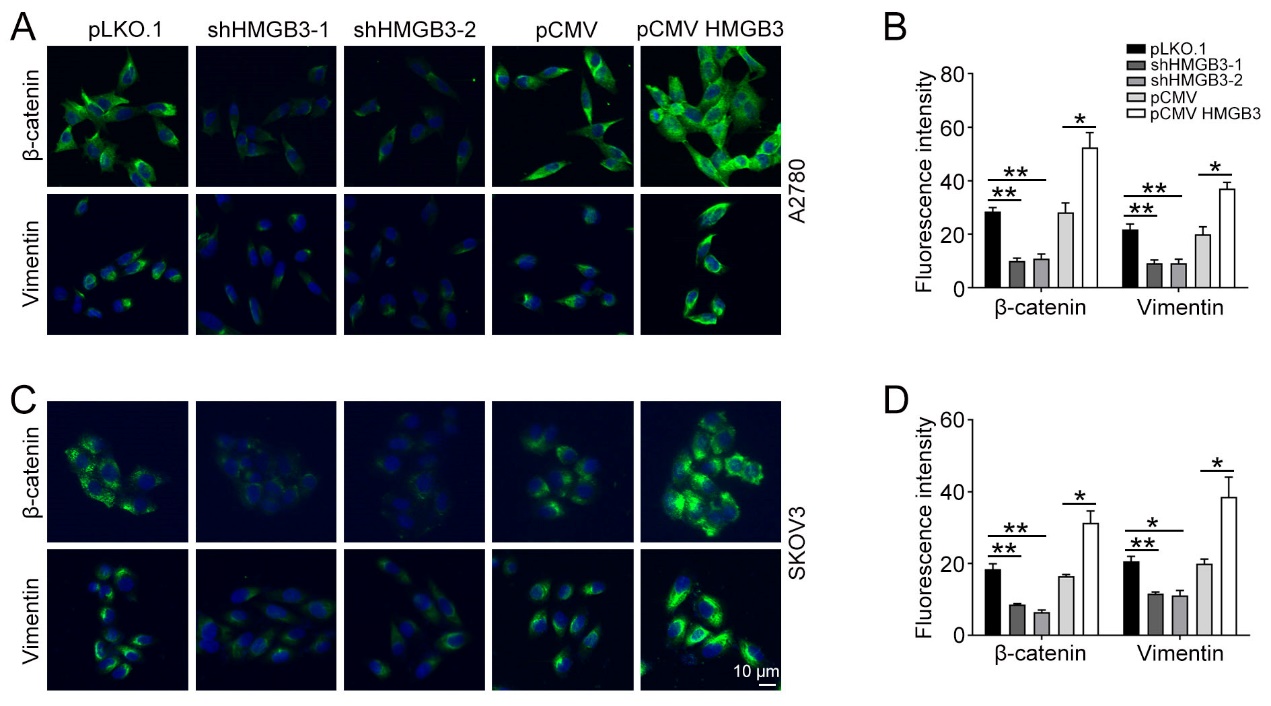


**Figure S2.** Immunofluorescence staining was performed to detect EMT-related markers in A2780 (A) and SKOV3 (C) cells with HMGB3 knockdown or overexpression. Nucleus was indicated by DAPI. (B) and (D) Quantification of the fluorescence intensity in (A) and (C). Scale bar, 10 µm. (Data are presented as the mean ± SEM, **p* < 0.05, ***p* < 0.01, n = 3).


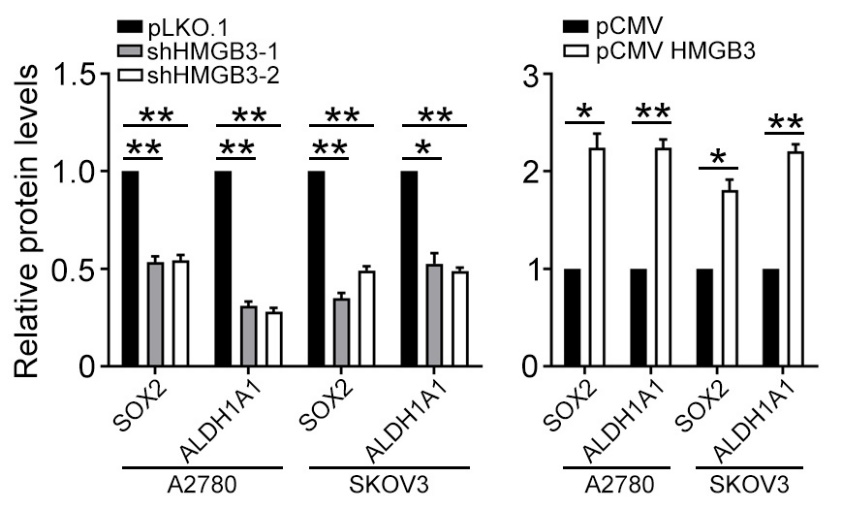


**Figure S3.** Quantification of the protein levels in Figure 5F. (Data are presented as the mean ± SEM, **p* < 0.05, ***p* < 0.01, n = 3).


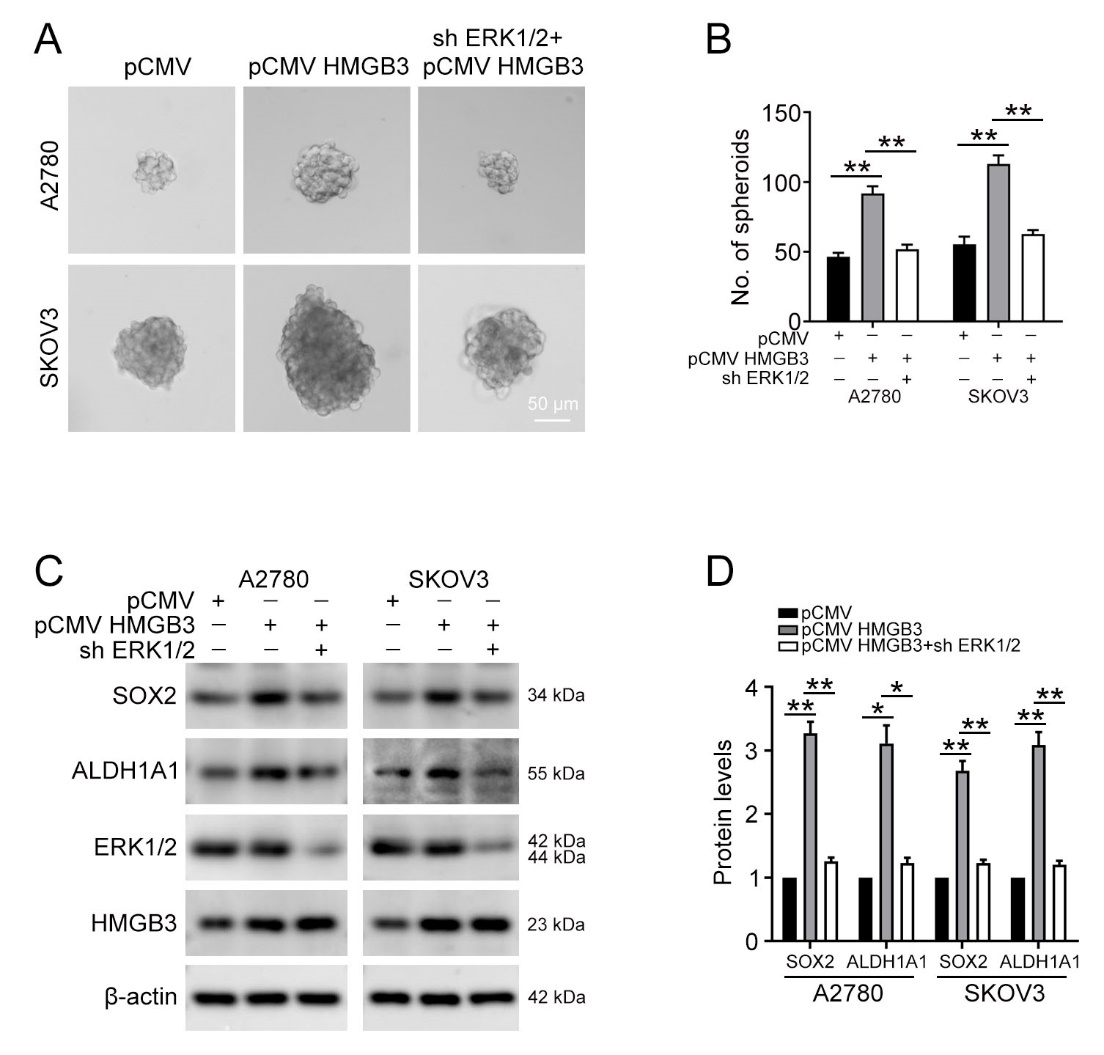


**Figure S4.** HMGB3 promotes the stemness of ovarian cancer by the MAPK/ERK signaling pathway. A2780 and SKOV3 cells stably transfected with pCMV, pCMV HMGB3, and/or ERK1/2 shRNA (sh ERK1/2) were cultured in semi-solid serum-free medium for 7 days. (A) The number and volume of spheroids formed were determined via microscopy, and representative pictures were shown. Scale bar, 50 µm. (B) Quantification of the number of spheroids in (A). (C) Western blot was performed to detect the protein levels of SOX2, ALDH1A1, ERK1/2, HMGB3, and β-actin in spheroids. (D) Quantification of the protein levels in (C). (Data are presented as the mean ± SEM, **p* < 0.05, ***p* < 0.01, n = 3).


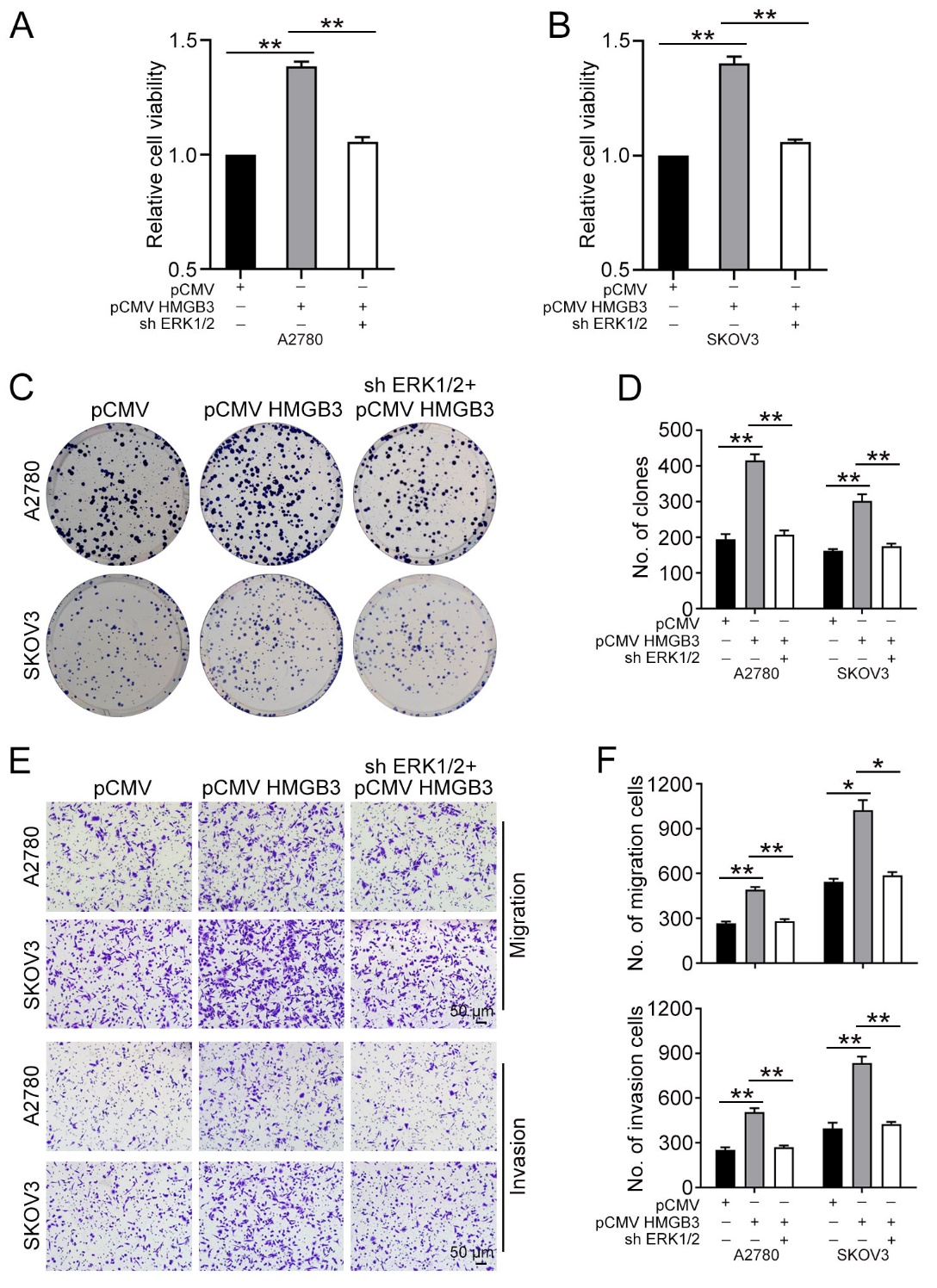


**Figure S5.** A2780 and SKOV3 cells were stably transfected with pCMV, pCMV HMGB3, and/or ERK1/2 shRNA (sh ERK1/2). (A) and (B) The MTT assay was performed to detect cell viability at 72 h. (C) The clonogenic assay was used to assess the colony formation efficiency of A2780 and SKOV3 cells. (D) Quantification of the number of clones in (C). (E) The Transwell assay was performed to determine cell migration and invasion of A2780 and SKOV3 cells. Scale bar, 50 µm. (F) Quantification of the number of cells in (E). (Data are presented as the mean ± SEM, ***p* < 0.01, **p* < 0.05, n = 3).


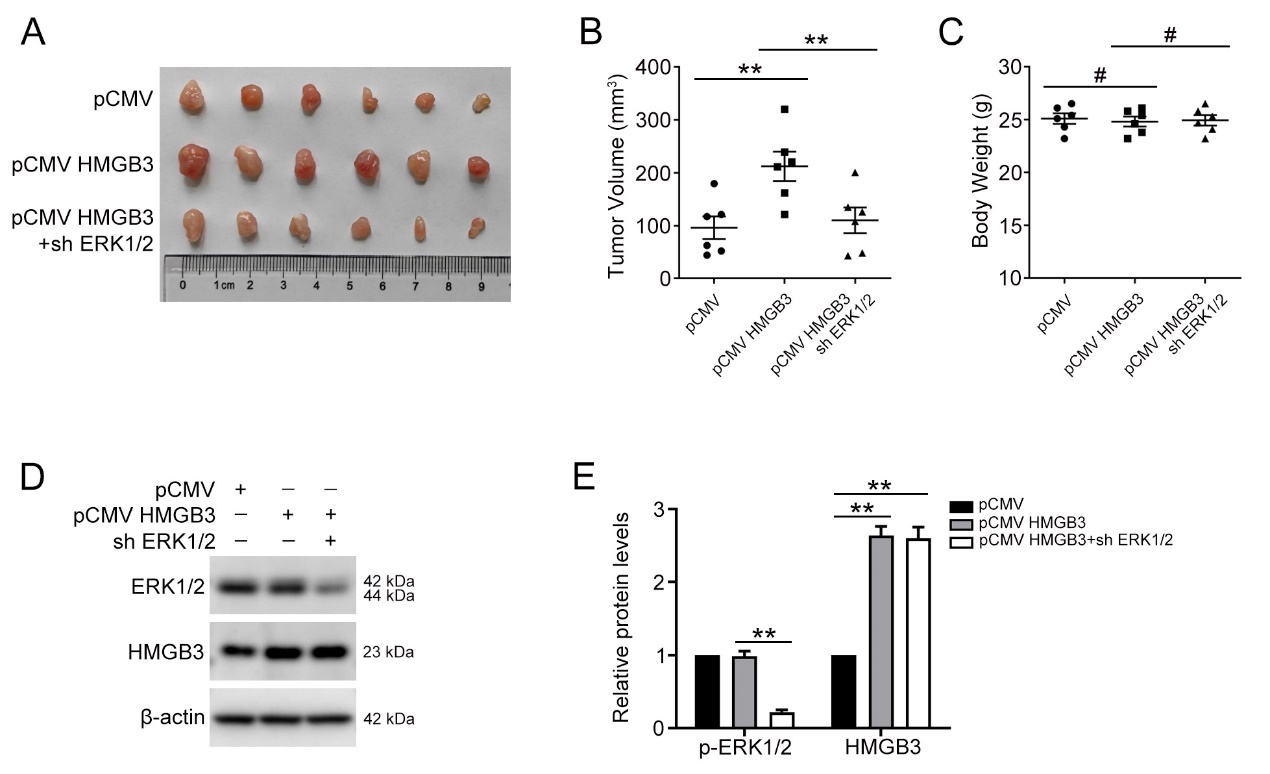


**Figure S6.** A2780 cells (5 × 10^6^) stably transfected with pCMV, pCMV HMGB3, and/or ERK1/2 shRNA (sh ERK1/2) were subcutaneously injected into nude mice. The mice were divided into three groups: pCMV (Ctr), pCMV HMGB3, and pCMV HMGB3+ sh ERK1/2. Each group contains six mice. Two weeks post-injection, the mice were euthanized and the xenograft tumors were removed. (A) Tumors from each group were shown. (B) The tumor volumes of each group. (C) The body weight of each group. (D) Western blot was used to detect the protein levels of ERK1/2, HMGB3, and β-actin in tumor tissues. (E) Quantification of the protein levels in (D). (Data are presented as the mean ± SEM, ^#^*p* > 0.05, ***p* < 0.01, n = 6).

**Supplementary Table S1**

siRNA sequences used for HMGB3 knockdown.

| **Gene** | **Sequences (5' to 3')** |
| --- | --- |
| HMGB3-siRNA | CUGUAUCAAAGUUGUACAU |

shRNA sequences used for ERK1/2 knockdown.

| **Gene** | **Sequences (5' to 3')** |
| --- | --- |
| ERK1/2-shRNA | AGCAATGACCACATCTGCTA |

**Supplementary Table S2**

**Primer sequences used for qRT-PCR**

| **Gene** | **Primer sequences (5' to 3')** |
| --- | --- |
| β-actin-F | CATGTACGTTGCTATCCAGGC |
| β-actin-R | CTCCTTAATGTCACGCACGAT |
| HMGB3-F | CCAAGAAGTGCTCTGAGAGGTG |
| HMGB3-R | CTTCTTGCCTCCCTTAGCTGGT |
| FGF18-F | ACGATGTGAGCCGTAAGCAGCT |
| FGF18-R | ACCGAAGGTGTCTGTCTCCACT |
| FGFR3-F | TCCATCTCCTGGCTGAAGAACG |
| FGFR3-R | TGTTCTCCACGACGCAGGTGTA |
| FGFR4-F | AACACCGTCAAGTTCCGCTGTC |
| FGFR4-R | CATCACGAGACTCCAGTGCTGA |
| HSPA1B-F | ACCTTCGACGTGTCCATCCTGA |
| HSPA1B-R | TCCTCCACGAAGTGGTTCACCA |
| MAP2K7-F | GACCTGGATGTGGTGCTGAAGA |
| MAP2K7-R | TCTTGAGCTTCTCAGCGCAGGT |
| MAP3K11-F | CTGGATGGCTCCTGAGGTTATC |
| MAP3K11-R | CACAGCAAGGCAGTCAATGCCA |
| MAPK8IP2-F | TCGTCAACAGCACATCTCGGTC |
| MAPK8IP2-R | CACCAACACAGGGTCATCCACA |
| NR4A1-F | GGACAACGCTTCATGCCAGCAT |
| NR4A1-R | CCTTGTTAGCCAGGCAGATGTAC |
| NTF4-F | GCAAGGCTGATAACGCTGAGGA |
| NTF4-R | CCTGGGCATCAGCGGTCAATG |
| RASGRP2-F | CTTCAACAGCGTCTCACAGTGG |
| RASGRP2-R | ACCACTGCCATCAGCGTGTTGA |
| RELB-F | TGTGGTGAGGATCTGCTTCCAG |
| RELB-R | TCGGCAAATCCGCAGCTCTGAT |
| TGFBR1-F | GACAACGTCAGGTTCTGGCTCA |
| TGFBR1-R | CCGCCACTTTCCTCTCCAAACT |
| APC2-F | GCCGACATCAACAGCAAGAAGG |
| APC2-R | CGCCTTGTTCTCTGTGCTGTGT |
| AXIN1-F | GTATGTGCAGGAGGTTATGCGG |
| AXIN1-R | CACCTTCCTCTGCGATCTTGTC |
| DVL1-F | GCATAACCGACTCCACCATGTC |
| DVL1-R | GATGGAGCCAATGTAGATGCCG |
| FZD2-F | TCGTGTACCTGTTCATCGGCAC |
| FZD2-R | CTGTGTAGAGCACGGAGAAGAC |
| ID3-F | CAGCTTAGCCAGGTGGAAATCC |
| ID3-R | GTCGTTGGAGATGACAAGTTCCG |
| TCF3-F | CCAGACCAAACTGCTCATCCTG |
| TCF3-R | TCGCCGTTTCAAACAGGCTGCT |
| N-cadherin-F | CCTCCAGAGTTTACTGCCATGAC |
| N-cadherin-R | GTAGGATCTCCGCCACTGATTC |
| Vimentin-F | AGGCAAAGCAGGAGTCCACTGA |
| Vimentin-R | ATCTGGCGTTCCAGGGACTCAT |
| β-catenin-F | CACAAGCAGAGTGCTGAAGGTG |
| β-catenin-R | GATTCCTGAGAGTCCAAAGACAG |
| Snail-F | TGCCCTCAAGATGCACATCCGA |
| Snail-R | GGGACAGGAGAAGGGCTTCTC |
| Slug-F | ATCTGCGGCAAGGCGTTTTCCA |
| Slug-R | GAGCCCTCAGATTTGACCTGTC |
